# The Substance of Cold: Indonesians’ Use of Cold Weather Theory to Explain Everyday Illnesses

**DOI:** 10.3389/fpsyg.2021.734044

**Published:** 2021-09-16

**Authors:** Florencia K. Anggoro, Benjamin D. Jee

**Affiliations:** ^1^Department of Psychology, College of the Holy Cross, Worcester, MA, United States; ^2^Department of Psychology, Worcester State University, Worcester, MA, United States

**Keywords:** folknatural theories, illness, culture, causal explanations, conceptual development

## Abstract

Many people across the world use cold conditions, such as cold air and wet clothes, to explain everyday illness, such as colds and flu. In Indonesia, the concept *masuk angin*, or “trapped wind,” appears to reflect this line of folknatural thinking. Interestingly, Indonesians distinguish masuk angin from the common cold, which is a frequent target for “cold weather” explanations in other cultures. We interviewed Indonesian 8- and 10-year-old children, lay adults, and medical expert adults, about the cause, contagiousness, and treatment of everyday illnesses: the common cold, the flu, and masuk angin. Most Indonesian children, and especially adults, believed that cold and flu are caused by germs and are contagious. In contrast, most children and lay adults (but not experts) attributed masuk angin to cold conditions and viewed it as non-contagious. These findings reveal how folknatural and scientific theories of illness coexist in the minds of Indonesian children and lay adults.

## Introduction

People’s causal beliefs about illness, from everyday colds to life-threatening diseases, guide their reasoning about health-related practices and policies. Cultural factors—the shared values, beliefs, practices, etc. that characterize a group of people—can also shape a person’s deeper conceptualizations of the entities and processes thought to cause illness. In many cultures, illness is attributed to *supernatural* causes, such as witchcraft and karma, or *religious* causes, such as God’s will (Legare and Shtulman, 2018). In South Africa, for example, children and adults often point to witchcraft—a curse directed at an individual or group—as a cause of HIV infection (Legare and Gelman, 2008). In the United States, about a third of adults have claimed to have “experienced or witnessed a divine healing of an illness or injury” (Pew Research Center, 2006).

Intuitions about nature and the physical world provide another framework for explaining illness (Legare and Shtulman, 2018). Children and adults often think of cold and heat as *substances* (e.g., Chi, 2005, 2012). However, heat—and its absence, cold—is a form of energy associated with the movement of invisible molecules. The intuitive notion of cold as a substance is integral to “cold weather theory,” a *folknatural* theory in which illness is directly attributed to cold conditions, e.g., “Don’t go out without a coat, or you may catch a cold.”

The idea that cold conditions lead to illness is widely accepted across cultures (Foster, 1976; Sigelman et al., 1993). In a US sample, most children between 6 and 9years of age asserted that cold weather can cause colds, and a sizeable proportion (around 50%) pointed to cold air as the causal agent (Sigelman, 2012). A majority of American and Mexican adult respondents agreed that colds are caused by exposure to drafts/wind/air and by not being properly clothed in cold weather (Baer et al., 1999). A study conducted in the 1970s in a suburb of London, England, found that adults often attributed colds to “cold forces” (e.g., damp, cold air) that penetrate the skin (Helman, 1978). In the Chinese tradition, the idea that cold causes illness manifests itself in a framework of hot/cold, yin/yang, and maintaining the balance of *qi*. Natural forces, such as high and low temperatures, rain, and wind, can disrupt the balance of *qi* and cause someone to fall ill. Chinese children, as young as kindergarten age, offer explanations of illness that involve “wind” and “cold” (Zhu et al., 2009). Hinton et al. (2001) document a well-articulated theory of wind overload in which the build-up of wind in the body is linked to a number of physical and psychological symptoms that can be debilitating or even deadly (Hinton et al., 2001). In Japan, the cold weather theory is connected to the concept of *vitalism*, the idea that living things are governed by internal/vital force. Exposure to cold air is believed to deplete a person’s internal “vital” force (Hatano and Inagaki, 1994).

In Indonesia, an ethnically diverse developing country with a population of 264 million people, cold weather theory is distilled into perhaps its purest form in the concept *masuk angin*, or “trapped wind.” The symptoms of *masuk angin* include headaches, nausea, and fever, and can range from mild fatigue to diarrhea and vomiting. There are numerous traditional remedies for *masuk angin*, including herbal remedies (known as *jamu*), which can be found in most Indonesian pharmacies and convenience stores. One such remedy is branded with the catch phrase, “reject the wind.” Other widely used treatments, such as cupping and skin scraping (*kerokan*), are intended to extract trapped wind through the skin’s surface.

Interestingly, *masuk angin* has not replaced the concept of the common cold. Indonesians have separate terms for “cold,” “flu,” and “trapped wind.” It is interesting to consider how children and adults conceptualize the three illnesses in light of this naming system. One possibility is that Indonesians will extend cold weather theory broadly, because their naming system—which includes separate terms for cold *and* trapped wind—places a disproportionate emphasis on cold conditions. On the other hand, it is possible that Indonesians will reserve their cold weather explanations for *masuk angin* alone, because the idea of wind as a causal agent is central to this particular illness. If *masuk angin* effectively appropriates the cold weather theory, then Indonesians may seek alternative causal explanations for cold and flu, perhaps grouping these two illnesses together in single explanatory framework.

The possibilities we have outlined—a broader extension of cold weather explanations on the one hand *vs*. a more restricted application on the other hand—may vary with age and, relatedly, familiarity with scientific ideas about illness. The use of cold weather theory appears to diminish as people learn how biological agents, such as viruses, cause illness (Helman, 1978; Au et al., 2008; Siegelman, 2014; Toyama, 2019). Thus, young children, with relatively little familiarity with germ theory, may be more likely to provide intuitive/folknatural explanations for the different illnesses. However, it is possible that cold weather theory will cognitively coexist in the minds of older children and adults, rather than being replaced outright by scientific ideas (Shtulman and Valcarcel, 2012; Legare and Shtulman, 2018; Kelemen, 2019; Hernandez et al., 2020). If so, will Indonesians incorporate cold and germ theories into a single explanation, or will they use different explanations for different illnesses?

The goal of the present study was to explore the interplay between intuitive and scientific ideas across different age groups and for three everyday illnesses: the common cold, the flu, and the culture-specific illness *masuk angin*. We conducted structured interviews with Indonesian children, lay adults with no medical background, and expert adults who were trained in Western medicine. We focus on 8- and 10-year-olds because at these ages, Indonesian children are likely to be familiar with the different types of illnesses (colds, flu, and the culture-specific *masuk angin*) and be able to explain their thinking. Our interviews probed the participant’s beliefs about the cause, contagiousness, and treatment of each illness.

## Materials and Methods

### Participants

Participants were 8-year-olds (*N*=25, *M_age_*=8.54years, 57% female), 10-year-olds (*N*=25, *M_age_*=10.49years, 56% female), novice adults (*N*=15, *M_age_*=42.23years, 73% female), and medical expert adults (*N*=13, *M* length at profession=11years, 85% female) recruited from Jakarta, Indonesia. Child participants were recruited from third- and fifth-grade classrooms at a private elementary school. Both novice and expert adult participants were recruited *via* word of mouth. The expert adults were physicians recruited from the same hospital in Jakarta. We do not have specific data on where they were trained, but our understanding is that Indonesian physicians typically have to be (re-)trained locally even if they have received medical education outside the country. Participants received a small non-monetary gift for their participation. Data were collected between 2013 and 2015.

### Materials and Procedure

Participants were tested individually in a quiet place in their school, campus, or workplace. The interview was conducted in the Indonesian language by a native speaker. Our first concern was whether the participant distinguished between the illnesses. We explicitly asked them, “Is a cold the same as the flu?” If cold and flu were not regarded as different by the participant, then the interviewer lumped these two illnesses together in their questions. The interview covered the cause, transmission, and treatment of each illness^1^. Participants were free to provide multiple responses, but were not explicitly prompted to do so. Table 1 contains the interview questions.

**Table 1:** Interview Questions.

1. What makes someone get [illness]? Can you tell me the ways in which someone could get [illness]?
2. For each causal factor, a. How would [the specific factor mentioned by the participant] give someone [illness]? b. If someone gets [illness] from [specific factor], does something get in their body that makes them get sick? What is it?
3. When you have [illness], do you seek treatment? If yes, what is it?
4. Is [illness] contagious? Can you give your [illness] to someone else? How does that happen?

### Interview Coding

Raw responses were translated to English by a native Indonesian speaker. Our coding focused on participants’ ideas about the cause and treatment of each illness. Participants’ causal explanations were coded using the following five categories: *germs* (e.g., a virus)*, cold conditions* (e.g., wind/cold air), *food ingestion* (e.g., eating oily foods), *unclean environment* (e.g., exposure to pollutants), and *physical vulnerability* (e.g., lack of sleep). Participants’ beliefs about treatment were coded using the following four categories: *medication* (e.g., over-the-counter and physician-prescribed medicine), *alternative treatment* (e.g., home remedies, alternative medicine, and alternative procedures), *health professional* (e.g., consulting a doctor or nurse), and *rest and nutrition* (e.g., sleep, hydration, and eating well). If the participant’s explanation included elements of multiple categories, we coded each category.

The first author coded the interview data. To establish the reliability of our coding scheme, the second author coded 15% of the interviews, including examples from each age and expertise group, randomly selected from the full data set. Inter-rater reliability was high (Cohen’s Kappa=0.84).

## Results

Many of the participants in our sample considered colds and flu to be the same: 76% of 8-year-olds, 64% of 10-year-olds, 54% of novice adults, and 69% of expert adults. For expediency, the interviewer asked these participants about the two illnesses together (e.g., what causes colds and flu?), under the assumption that their responses would be redundant if asked about the illnesses separately. In such cases, the same response was filled in for the cold and flu section of the interview. Our general analytic strategy was to compare the frequency with which a particular type of response was expressed by members of each of our four groups of participants using chi-square analyses.

### Causal Explanations

**Figure 1:**
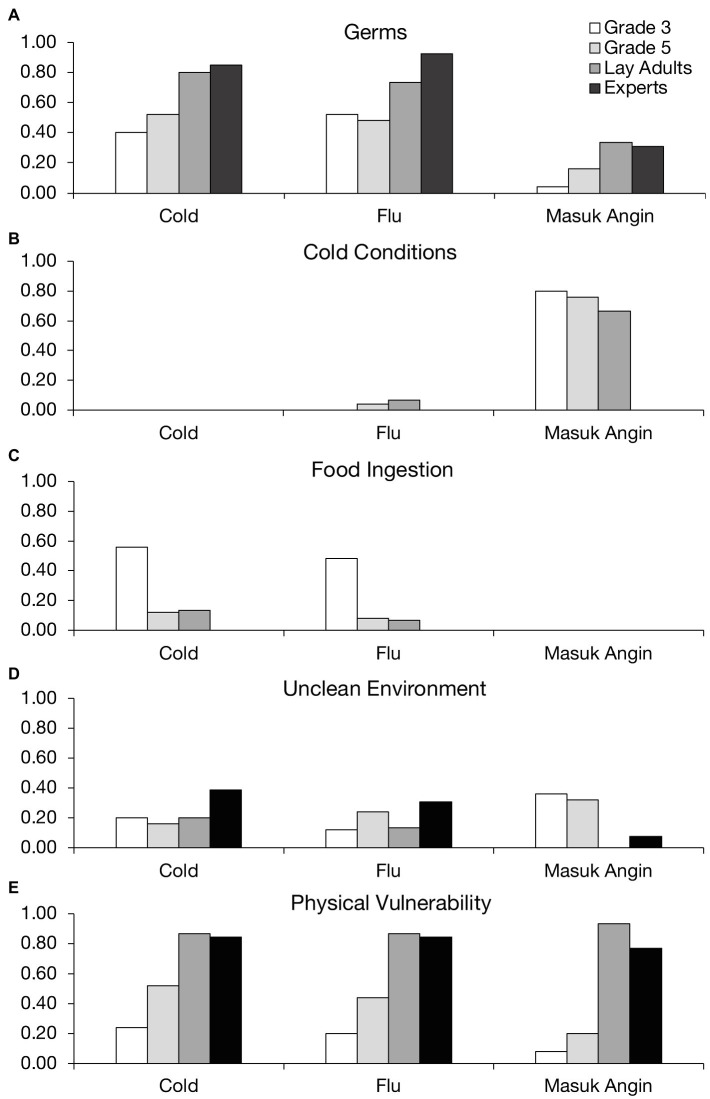
Proportion of participants who mentioned a given cause for each illness.

Figure 1 shows the proportion of participants from each age/expertise group who mentioned a given cause (*germs, cold conditions*, *food ingestion*, *unclean environment*, and *physical vulnerability*) in their explanations of cold, flu, and *masuk angin*.

From Figure 1A, it is clear that, overall, participants mentioned germs more often when explaining cold and flu compared to *masuk angin*. Lay adults and medical experts were more likely than 8- and 10-year-old children to invoke germ theory in their explanations of cold, *χ*^2^(3, *N*=78)=10.50, *p=0*.01, and flu, *χ*^2^(3, *N*=78)=8.98, *p=0*.03. For *masuk angin*, however, even older participants—who clearly were aware of germ theory—seldom mentioned germs in their explanations. Instead, we can see from Figure 1B that the vast majority of children and lay adults relied on cold conditions to explain *masuk angin*, although not surprisingly, none of the medical experts did so, *χ*^2^(3, *N*=78)=23.98, *p*<0.0001. Interestingly, children’s and adults’ use of cold weather theory was largely restricted to *masuk angin*. For cold and flu, cold conditions were almost never mentioned as a cause, even by children in the youngest age group.

Figure 1C sheds more light on younger children’s causal beliefs about cold and flu. Food ingestion was mentioned as a cause of these illnesses by about half of the 8-year-olds in our sample, a significantly higher proportion than the other groups, *χ*^2^(3, *N*=78)=17.21, *p*<0.001 for cold, and *χ*^2^(3, *N*=78)=15.67, *p*=0.001 for flu. A moderate proportion of children from each age group mentioned unclean environment as a cause of each illness (Figure 1D), though there were no significant age or expertise-related differences for this factor, *χ*^2^(3, *N*=78)=2.69, *p*=0.44 for cold, *χ*^2^(3, *N*=78)=2.66, *p*=0.45 for flu, and *χ*^2^(3, *N*=78)=7.36, *p*=0.06 for *masuk angin*. A more interesting pattern emerged for *physical vulnerability* (Figure 1E). Although a moderate number of children, especially 10-year-olds, mentioned this factor, a higher proportion of lay adults and medical experts—almost all of them, in fact—cited physical vulnerability as a cause of each illness, *χ*^2^s(3, *N*=78)>20.00, *p*s<0.0005.

The emerging picture is that Indonesian adults tend to view illness as a byproduct of physical vulnerability plus exposure to either germs (in the case of cold and flu) or cold conditions (in the case of *masuk angin*). To further explore this pattern, we classified each participant according to whether they mentioned a single causal factor in their explanation for each illness or a combination of factors, focusing on the most common causes: germs, cold conditions, and physical vulnerability. We then performed chi-square analyses on the frequency of single-cause and multi-cause explanations for each illness from each group of participants. Most lay adults attributed illness to a combination of factors (either germs or cold conditions plus physical vulnerability), as opposed to one factor alone, *χ*^2^s(3, *N*=15)>11.5, *p*s<0.01. The medical experts show a similar pattern, though they never mentioned cold conditions. Eight-year-olds, however, tended to point to a single cause: especially, germs in the case of cold, *χ*^2^(3, *N*=13)=7.62, *p*=0.05, and flu, *χ*^2^(3, *N*=16)=17.50, *p*<0.001, and cold conditions for *masuk angin*, *χ*^2^(3, *N*=21)=41.67, *p*<0.00001. Ten-year-olds were more likely to incorporate physical vulnerability, but still favored single-cause explanations, especially for *masuk angin*, *χ*^2^(3, *N*=24)=27.67, *p*<0.00001. Thus, with increasing age and experience comes a greater realization that illness is affected not only by outside agents, but also the human body’s ability/inability to defend itself, especially in the case of germ-based illnesses.

### Transmission

**Figure 2:**
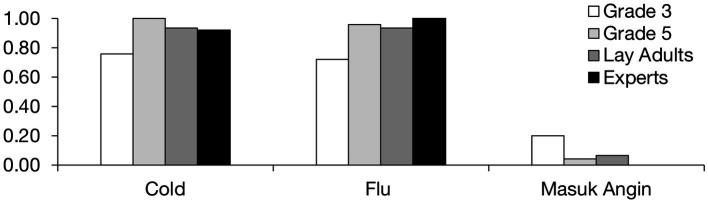
Proportion of participants who asserted that each illness is contagious.

Our next analyses concern participants’ beliefs about whether a given illness can be transmitted from one person to another. Figure 2 shows the proportion of participants from each age/expertise group who asserted that cold, flu, and *masuk angin* could spread from one person to another. We conducted chi-square analyses to compare the frequency of these affirmative responses between groups for each illness. The majority of participants believed in person-to-person spread for cold and flu. Although 8-year-olds were least likely to mention this form of contagion, there were no significant age or expertise-related differences, *χ*^2^=(3, *N*=78) 5.73, *p*=0.13 for cold, and *χ*^2^(3, *N*=78)=7.71, *p*=0.05 for flu. Participants’ beliefs about *masuk angin* were strikingly different. Almost none of the participants agreed that *masuk angin* can spread from one person to another. There was no significant difference between the groups, *χ*^2^(3, *N*=78)=3.94, *p*=0.27. Altogether, these findings complement what we found with participants’ causal explanations. The illnesses that were often attributed to germs (colds and flu) were viewed as contagious, whereas the illness that was attributed to cold conditions (*masuk angin*) was not.

### Treatment

**Figure 3:**
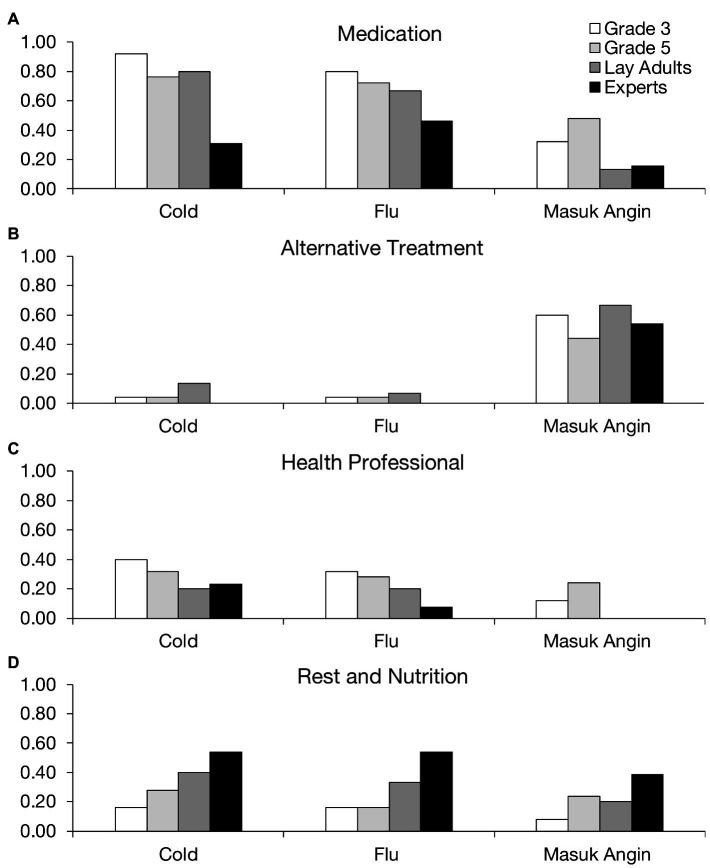
Proportion of participants who mentioned a given treatment for each illness.

Finally, we consider participants’ beliefs about how the different illnesses can be treated. Figure 3 shows the proportion of participants who mentioned a given type of treatment (*medication*, *alternative treatment*, *health professional*, and *rest and nutrition*) for each illness. We compared the frequency with which these treatment types were mentioned by each group of participants for each illness separately.

Figure 3A shows that participants frequently mentioned medication, especially as a treatment for cold and flu. In fact, children and lay adults were more likely than medical experts to endorse medication as a treatment for colds, *χ*^2^(3, *N*=78)=17.3214, *p*=0.0006, although this trend was not significant for flu, *χ*^2^(3, *N*=78)=4.75, *p*=0.19. Children also tended to mention medication to treat *masuk angin*, although the trend was not significant, *χ*^2^(3, *N*=78)=7.09, *p*=0.07. Figure 3B shows that alternative treatments, including herbal remedies and skin scraping, were a preferred remedy for *masuk angin*. About the same proportion of participants from each group—around half—mentioned alternative treatments for *masuk angin*, *χ*^2^(3, *N*=78)=2.31, *p*=0.51. Such treatments were seldom mentioned for cold and flu, however.

A moderate proportion of participants mentioned treatment from a health professional (Figure 3C). Perhaps, surprisingly, medical experts were no more likely than the other groups to mention this treatment for any illness, *χ*^2^s(3, *N*=78)<4.50, *p*s>0.20. Figure 3D shows that the expert participants were the most likely to recommend rest and nutrition as a treatment, especially for the flu, *χ*^2^(3, *N*=78)=8.33, *p*=0.04. There were similar results for the other illnesses, although the trends were not significant for cold, *χ*^2^(3, *N*=78)=6.50, *p*=0.09, and *masuk angin*, *χ*^2^(3, *N*=78)=5.16, *p*=0.16.

Overall, the results revealed a general tendency for participants to treat cold and flu with medication, and *masuk angin* with alternative treatments. Even the younger participant groups showed this pattern, although they tended to also mention medication for *masuk angin*. Perhaps, this was because the distinction between medication and alternative treatments was not as salient to the children as it was to the adult groups. Interestingly, the medical experts did not dismiss alternative treatments for *masuk angin* and endorsed these treatments about as often as the other groups. Thus, although experts disagreed that cold conditions cause *masuk angin*, they did regard alternative treatments as an effective course of treatment.

## Discussion

Indonesian children and adults tended to express similar ideas about cold and flu; namely, that these illnesses are caused by germs, are contagious, and are treatable by medication. With age, participants were more likely to invoke germ theory for these illnesses, and to incorporate physical vulnerability as a contributing factor. Ideas about *masuk angin* were strikingly different. Indonesian children and adults tended to attribute *masuk angin* to cold conditions, to view the illness as non-contagious, and to point to alternative medicines and practices as effective treatments. Medical experts did not endorse cold weather theory for *masuk angin*, but mentioned alternative treatments about as often as the other groups.

Our results imply that scientific and folknatural theories of illness coexist in the minds of many Indonesian children and lay adults. Most participants mentioned both germs *and* cold conditions as causal factors. Rather than blending cold weather and germ theories in their explanations, however, cold weather theory was reserved for *masuk angin* alone, a form of “target-dependent” reasoning^2^ (Legare and Shtulman, 2018). Even for colds—which children and lay adults in other parts of the world often explain at least in part with cold conditions (Helman, 1978; Baer et al., 1999; Sigelman, 2012; Hernandez et al., 2020)—Indonesians seldom mentioned cold as a cause. Thus, *masuk angin* appears to have fully appropriated the cold weather theory. This could help explain why many participants regarded cold and flu as essentially the same condition: The differences between cold and flu may seem trivial in light of the deeper distinction between germ- and cold-based illness.

For the most part, the participants expressed causally coherent ideas about the different illnesses. The illnesses that were attributed to germs (cold and flu) were also viewed as contagious and treatable with medication. The illness that was attributed to cold conditions (*masuk angin*) was viewed as non-transmissible and treatable with alternative treatments, such as herbal remedies that release the “trapped wind.” Yet, despite this internal consistency, cold weather and germ theories are scientifically incompatible—indeed, Indonesian medical experts gave no credence to the notion of cold as a cause of illness. It is possible that the children and lay adults were not aware of the conflict, as is sometimes the case with coexisting beliefs (Legare and Shtulman, 2018). Traditional beliefs are widespread in Indonesia (Cochrane, 2018), potentially blurring the boundary between folk and scientific ideas. The extent to which people seek explanations and attempt to resolve conflicts also varies across cultures (Legare et al., 2013). Even if an inconsistency is detected between cold weather and germ theories, Indonesia’s cultural emphasis on harmony and finding a “middle way”—a common mode of reasoning among East Asians (Nisbett et al., 2001)—may encourage people to avoid a single answer.

One interesting age-related pattern was that older children and adults tended to mention the body’s vulnerability as an important causal factor in illness. This could signify a progression from viewing the body as being passively affected by outside agents to actively counteracting those agents. Our participants seem to hold fairly broad ideas about the body’s vulnerability, often describing it in terms of a general level of stamina or energy. One interesting question is how participants view the body’s response to different agents, such as a virus *vs*. the wind. Another question is how participants think the body might develop immunity from an infection or respond to vaccinations. These causal beliefs may be related to people’s attitudes about vaccination and other preventative measures, such as masking and social distancing.

There are several limitations to the present research. Our participants were recruited from Indonesia’s largest city, Jakarta. Regional differences in lifestyle, education, access to medical facilities, etc., may be associated with differences in children’s and adults’ conceptions of illness, including *masuk angin*. Furthermore, our interview questions, though open-ended, did not delve into participants religious or spiritual beliefs about the cause and treatment of illness. Such beliefs are widespread and may coexist with the kinds of causal explanation we have described in the present work (Legare and Gelman, 2008; Legare and Shtulman, 2018). Our study was also limited to common, everyday illnesses and did not explore beliefs about other, more serious conditions, like cancer, or mental illnesses, like depression. Expanding the scope of our interviews would shed light on how folknatural and other kinds explanations are used across a range of illness types. Another issue to explore is how the use of folknatural explanations for illness relates to causal explanations in *other* domains. Perhaps, thinking of cold as a substance (an ontological error; Chi et al., 2012) is part of a broader tendency to accept intuitive explanations in chemistry, physics, space science, and other fields. Indeed, the tendency to rely on intuition over reflective analytic thinking predicts poorer understanding of basic ideas in science (Young and Shtulman, 2020).

The participants in our study generally agreed that cold and flu are contagious, but *masuk angin* is not. This distinction could have significant consequences for how Indonesians behave when ill. With *masuk angin*, there is no need to avoid others when sick, nor is there a need to take precautions when another person is sick. If the illness in question actually turns out to be a cold or flu, this *laissez-faire* response could increase the spread of infection. Thus, a lot rests on how an illness is diagnosed. In the future research, it will be informative to test how children and adults determine a diagnosis from a given set of symptoms, e.g., in a description of a hypothetical patient. It will also be interesting to explore how people’s thinking about a case is affected when a diagnostic label is provided along with it. Labels have a powerful influence on children’s and adults’ reasoning, and can override feature-based similarity when category membership and similarity are in competition (Yamauchi, and Markman, 2000; Gelman, 2004). Perhaps, labeling an illness as *masuk angin* will lead people to ignore important symptoms and risk factors that may otherwise guide their response.

Although we focused on children’s and adults’ conceptual representations of everyday illnesses, these conceptualizations may be rooted in deeper ideologies and values (e.g., Hornsey and Fielding, 2017). Indonesians place a high value on interpersonal relationships and group harmony, as is common in East Asian relational-collectivist societies (Brewer and Chen, 2007). Thus, Indonesians may be motivated to classify an ambiguous and minor illness as *masuk angin*, a non-contagious illness, in part out of a desire to maintain social cohesion. The calculus may change for more serious, life-threatening conditions in which the risk of spreading the illness outweighs the benefit of maintaining social harmony. To this point, during the COVID-19 pandemic, people in more collectivist countries adopted preventive measures more widely and willingly than those in more individualistic countries (Maaravi et al., 2021). This same worldview was displayed by so-called “anti-maskers” in the United States, who protested mask use during the COVID-19 pandemic with slogans such as, “My Child, My Choice,” and “By Choice, Not Force.” (Fisher, 2020). People who prioritize individual freedom tended to oppose collective actions and preventive measures to combat the disease (Bazzi et al., 2021; Clarke et al., 2021). Similarly, vaccine hesitancy—a person’s refusal or delay of some vaccines for themselves or their children (World Health Organisation, 2019)—has links to cultural, religious, and political ideologies (Larson et al., 2014). For example, people whose cultural worldview emphasizes individualism and hierarchical social structure tended to judge a mandatory HPV vaccination program as relatively high in risk, because such programs are seen to challenge gender norms and infringe on individual choice (Kahan et al., 2010). In fact, individualistic/hierarchical worldview was among the four strongest predictors of anti-vaccination attitudes in a large-scale multinational study (Hornsey et al., 2018).

## Conclusion

Our research sheds light on how Indonesian children and adults conceptualize everyday illnesses, including the culture-specific illness of *masuk angin*. Cold conditions were frequently mentioned as a cause of illness, but were largely restricted to cases of *masuk angin*. This pattern was evident in our youngest age group (8-year-olds) and in adults. Beliefs about contagion and treatment were linked to participants’ causal explanations, highlighting the role of folknatural concepts in health-related behaviors. The depth and scope of culture-specific folknatural thinking is an important area for future research.

## Data Availability Statement

The raw data supporting the conclusions of this article will be made available by the authors, without undue reservation.

## Ethics Statement

The studies involving human participants were reviewed and approved by the Institutional Review Board, College of the Holy Cross. Written informed consent to participate in this study was provided by the participants’ parents or legal guardians.

## Author Contributions

FA managed data collection, coded all the data, with BJ coding a subset for reliability, and wrote the first draft of the manuscript. BJ provided the critical revisions. FA and BJ conceptualized the idea for the project, conducted the statistical analyses, interpreted the findings, outlined the manuscript, and approved the final version of the manuscript for submission.

## Funding

This research was supported by a Research and Publication Grant from the College of the Holy Cross.

## Conflict of Interest

The authors declare that the research was conducted in the absence of any commercial or financial relationships that could be construed as a potential conflict of interest.

## Publisher’s Note

All claims expressed in this article are solely those of the authors and do not necessarily represent those of their affiliated organizations, or those of the publisher, the editors and the reviewers. Any product that may be evaluated in this article, or claim that may be made by its manufacturer, is not guaranteed or endorsed by the publisher.

## Footnotes

^1^These questions were part of a larger protocol that included questions about illness prevention and lifestyle

^2^Even though this term has been used to refer to reasoning about a single event, we find it is appropriate to describe Indonesians’ reasoning as target-dependent given that the event in question is culturally shaped and contextually dependent.
